# Supplementary material for: Research on measurement indexes and evaluation for the collaborative efficiency of emergency information sharing in coastal cities of China
Source: PLoS One. 2022 Dec 1;17(12):e0276589. doi: 10.1371/journal.pone.0276589 (PMC9714706; doi:10.1371/journal.pone.0276589)
Supplement: Attachment — Submitted filename: Author response.docx [file pone.0276589.s001.docx]

Thank the reviewer for providing the direction for the author to improve the manuscript. According to the reviewer's suggestions, the author has made the modifications and explanations to the manuscript:

1. Introduction: i) What are the emergency public emergencies? It is necessary to demonstrate the difference between coastal cities and inland cities. ii) How can the establishment of an institution (National Emergency Management Department) “completely” change the pattern? iii) The lack of comparison between other countries' coastal cities in the same period, otherwise, it is not appropriate to directly say that China's low level. iv) Literature reviews should be classified based on research content. v) I don't think the existing research lacks research on the emergency mechanism of coastal cities, given that there are so many coastal cities in the world. Or what is special to the coastal city emergency management system.

**Author response:**

i) Public emergencies refer to negative events that occur unexpectedly under the domination of certain natural factors, cause serious harm, loss or impact to the society and need to be dealt with immediately. At the beginning of this paper, the special features of coastal cities and their special factors for disasters are proposed.
ii) In this paper, the "completely changing model" is changed to emphasize the improvement of emergency management capabilities.
iii) The gap between China's coastal cities and foreign countries can be seen from the literature review by foreign scholars on the emergency management capabilities of coastal cities.
iv) The literature review is organized from the aspects of emergency information sharing collaborative research, emergency information sharing mechanism research and emergency collaborative governance.
v) Firstly, the research of this paper is based on the theory of collaborative governance. Secondly, this paper proposes a framework and operation mechanism for coastal cities' emergency information sharing based on the collaborative theory, and proposes an index system and measurement method that affects the collaborative efficiency of coastal cities' emergency information sharing. This paper provided support for disaster prevention and mitigation information sharing decision-making.

2. Emergency Information Sharing Framework Based on Collaboration Theory: i) Are the four elements of the coastal city emergency information sharing framework original? The name of the “information participants” is not appropriate, and it contains too much content. ii) As previously written, “The emergency response department is mainly responsible for executing the emergency tasks and its responsibilities issued by the command center”. Information collection is not its responsibility. And how to conclude that “the emergency response department is not active, relying on the decision and instructions of the emergency command center, and the efficiency of information collection, disposal and feedback is low.”

**Author response:**

i) The four elements of the coastal city emergency information sharing framework are original. Emergency management information involves the coordination of various government departments. The research in this paper includes all relevant departments as much as possible.
ii) The main responsibility of the emergency response department is indeed not information collection, but before it orders tasks on its own, it needs to adjust its own response strategy in time based on the information collected. The conclusions drawn in this paper are based on the relevant literature and research results.

3. Coastal city emergency information sharing collaborative efficiency measurement: Whether this Coastal city emergency information sharing collaborative efficiency measurement is common to coastal cities and inland cities?

**Author response:** The collaborative efficiency measure of emergency information sharing in coastal cities is an influencing factor refined based on the characteristics of coastal cities and the theory of collaborative governance. Therefore, it is general for coastal cities, but not suitable for inland cities.

4. Empirical analysis: “The overall efficiency of China's coastal cities' emergency information sharing is low”, This indicator is low in the world, and it is still relatively low compared to inland cities?

**Author response:** This metric is lower worldwide, not just within China.

5. Editorial errors: i) “Basic information resources include five aspects: politics, economy, social development, resources, environment, and geographic information”, This statement is incorrect. ii) The words in the text box in Figure 2 should be left intact and a hyphen is required for line breaks. iii) In the “Policy suggestion”, at the end of the second suggestion is the fourth.

**Author response:**

i) Resources and environment are not two words, but one word reflecting two aspects, and the author has corrected the English translation.

ii) The author has adjusted the formativeness of the graph.

iii) The author changed (4) to (3)

In addition to making revisions based on the above suggestions, we have also improved the grammatical structure of the manuscript. We hope that reviewers can make further suggestions for revisions. Thank reviewer again!
