# Supplementary material for: Research on measurement indexes and evaluation for the collaborative efficiency of emergency information sharing in coastal cities of China
Source: PLoS One. 2022 Dec 1;17(12):e0276589. doi: 10.1371/journal.pone.0276589 (PMC9714706; doi:10.1371/journal.pone.0276589)
Supplement: Attachment — Submitted filename: Response to Reviewers.docx [file pone.0276589.s002.docx]

**Response to Reviewers**

Reviewer #2:

**Question 1:** Introduction and Discussion: The manuscript fails to address how the findings relate to previous research in this area. In addition, 22 of 28 references regard Chinese case studies. It seems that the proposed study regards only this specific geographic position. The authors should rewrite their “Introduction” and add “Discussion” to reference more related literature, especially recently published international work. The authors should also elaborate better the originality and novelty of their study, commenting a bit more on the representativeness and the generalizability of the study to broader socioeconomic contexts.

**Author response:** Author adds the following contents in the part of introduction to increase to increase foreign literatures : Carminati B et al(2016)^[14]^ presented an emergency information sharing framework able to deal with both specified and unspecified emergencies; Dantas A A et al(2007)^[15]^ presented an information-sharing framework for road organizations, on the basis of a study of response and recovery activities, information needs were identified and a geographic information system-based information-sharing framework was created.

Author adds the following contents in the part of introduction to increase to find relate to previous research in this area and to explain this innovation: The relationship between this paper and the previous research lies in: First, the index system of the existing research is optimized, and an effective evaluation index system is put forward from three dimensions, included the mechanism construction level of emergency information sharing, the resource support capability and the collaborative driving force. Secondly, the coupling theory in physics is used for reference to quantitatively evaluate the coordination efficiency of emergency information sharing in coastal cities. Thirdly, referring to the existing emergency information organization structure, the paper puts forward the emergency information sharing framework of China's coastal cities based on collaboration theory. The difference of this paper is that it proposes a coastal city emergency information sharing framework and operation mechanism based on the synergy theory, and proposes an index system and measurement method that affect the coastal city emergency information sharing collaboration efficiency.

Author also adds some references, as follows:

## [14] [Carminati B](https://www.webofscience.com/wos/alldb/general-summary?queryJson=%5B%7B%22rowBoolean%22:null,%22rowField%22:%22AU%22,%22rowText%22:%22Carminati,%20Barbara%22%7D%5D&eventMode=oneClickSearch), [Ferrari E](https://www.webofscience.com/wos/alldb/general-summary?queryJson=%5B%7B%22rowBoolean%22:null,%22rowField%22:%22AU%22,%22rowText%22:%22Ferrari,%20Elena%22%7D%5D&eventMode=oneClickSearch), [Guglielmi M](https://www.webofscience.com/wos/alldb/general-summary?queryJson=%5B%7B%22rowBoolean%22:null,%22rowField%22:%22AU%22,%22rowText%22:%22Guglielmi,%20Michele%22%7D%5D&eventMode=oneClickSearch), 2016, Detection of Unspecified Emergencies for Controlled Information Sharing. *IEEE Transactions on Dependable and Secure Computing*, 13(6):630-643.

## [15] Dantas A A, Sevile E and Gohil D,2007, Information sharing during emergency response and recovery - A framework for road organizations, *Transportation Research Record*, 2022,21-28.

[24] Kwon T. H, Zmud R. W., 1987, Unifying the fragmented models of information systems implementation, critical Issues in Information Systems Research. *New York: John Wiley*: 135 -142.

[25] Varun Grover, Martin D. Goslar, 1993, The initiation, adoption, and implementation of telecommunications technologies in U.S. organizations. *Journal of Management Information Systems*, 10(1): 141-163.

**Question 2:** Section 3: Coastal city emergency information sharing collaborative efficiency measurement. This study requires more conceptual details on the choice of the indicators and methods. The authors should include more information that clarifies and justifies their choice of methods. In addition, it is better to elaborate more on the meaning of the indicators, the gap and implication for the evaluation system taking the previous example for better understanding.

**Author response:** Author adds some contents in the part of section 3, as follows:

(1) *Principles of index selection*

*1)Systematization principle*

Indexes can reflect both direct and indirect effects, and ensure the comprehensiveness and credibility of comprehensive evaluation.

*2) Principle of index measurability*

The meaning of indexes is clear, the data required for calculating indexes are easy to collect, and the calculation method is simple and easy to master.

*3) Principle of correlation between indexes and targets*

The realization of indexes must make a substantial contribution to the realization of the goal, and it is forbidden to choose indexes unrelated to the goal.

Author also adds some contents about obtaining original data of indexes, as follows:

*4) Method of obtaining original data of indexes*

As most of the measurement indexes are qualitative, it is difficult to obtain data through monitoring equipment, so the method of expert assignment value is adopted to obtain the original data, and five levels of quantity are adopted to assign values to each measurement indicator: good =5, good =4, fair =3, poor =2 and poor =1.

**Question 3:** Section 4: Empirical analysis. 13 disaster events that occurred in coastal cities in China in the past 20 years are selected as samples. On what basis the authors selected those events as samples and representativeness of these samples should be more elaborate. Additionally, it was mentioned in the paper that contributions of U1, U2 and U3 subsystem had risen steadily. However, the presented data result in Table 4 does not fully support the conclusion. The authors should check carefully and confirm that the conclusions are consistent with the research results, and the recommendations are corresponding to the conclusions.

**Author response:** Author adds some contents in the part “Empirical analysis”, as follows:

The reason for choosing these samples is that these samples represent almost all the characteristics of coastal cities in China, and they are all major marine disasters. These sample data are relatively complete and have been collected on.

The author modified some contents in order to keep Table 4 consistent with the conclusion, as follows:

*（2）Although the contribution level of each subsystem has experienced twists and turns, it has generally increased.*

The 13 events in Table 4 are arranged in chronological order. The calculation results show that, on the whole, the contribution levels of the subsystems U1= emergency information sharing mechanism construction level, U2= emergency information sharing resource support capability and U3= emergency information sharing collaborative driving force are obviously increasing, which indicates that with the improvement of disaster prevention and mitigation capability of coastal cities in China, the collaborative capability of emergency information sharing is also increasing year by year.

**Other minor reviews:**
i) The statement in paragraph 1 of Section 1 and Section 2.3 should be justified by citations.

Author response: The author has cited the literature according to the expert opinion.

ii) Emergency information participants and emergency information resources are in a parallel relationship. The presentation of the relationship between them in Figure 1 seems to be an inclusive relationship, which is obviously inappropriate.

**Author response:** The author has modified Fig. 1 according to expert opinions, as shown in the following figure.

iii) Figure 3 is mentioned in Section 2.3 (1). However, there is no Figure 3 in the revised manuscript.

**Author response:** The author has modified Figure 3 to Figure 2.

iv) Is it emergency efficiency or collaborative efficiency in the title of Section 3?

**Author response:** It is emergency collaborative efficiency. Author had modified the title.

v) Please add the year information for the time of occurrence in Table 3.

**Author response:** Author had increased the year in Table 3.

vi) There are some typos and grammar mistakes in this manuscript. Please carefully check and correct these mistakes. Furthermore, moderate revisions are requested in order to polish the language usage (longer sentences and technicalities).

**Author response:** Author had edited some typos and grammar mistakes to this manuscript again.

vii) Please make sure all dash, space, a hyphen, dash, and capital words would be appropriate throughout the manuscript.

**Author response:** The author proofread the whole manuscript again.

viii) Please ensure that the font size and capital letters are consistent in the table and figure.

**Author response:** Author proofread to the font size and capital letters in the table and figure.

**Sincerely thank editors and peer experts for their suggestions!**
