# Supplementary material for: Research on measurement indexes and evaluation for the collaborative efficiency of emergency information sharing in coastal cities of China
Source: PLoS One. 2022 Dec 1;17(12):e0276589. doi: 10.1371/journal.pone.0276589 (PMC9714706; doi:10.1371/journal.pone.0276589)
Supplement: Attachment — Submitted filename: Response to Reviewers.docx [file pone.0276589.s004.docx]

**Response to Reviewers**

**Journal Requirements:**

Please review your reference list to ensure that it is complete and correct. If you have cited papers that have been retracted, please include the rationale for doing so in the manuscript text, or remove these references and replace them with relevant current references. Any changes to the reference list should be mentioned in the rebuttal letter that accompanies your revised manuscript. If you need to cite a retracted article, indicate the article’s retracted status in the References list and also include a citation and full reference for the retraction notice.

**Author response**: Author reviewed the reference list to ensure that it is complete and correct again.

**Additional Editor Comments:**

Although reviewer 1 has accepted the current version reviewer 2 raised some crucial grammatical mistakes and textual errors. The reviewer also suggested elaborating by providing a detailed discussion of the reasons for choosing some existing results in the proposed index for measuring the efficiency of emergency information sharing collaboration. Therefore, we would like to invite you to submit a revised version of the manuscript by correcting those errors and issues raised and also a detailed response to reviewer 2.

**Author response**: Author invited scholars from English-speaking countries to update the grammatical errors and text errors of this manuscript again. The revised contents in the manuscript are marked in red.

Author added some contents in 3.1 section to provide a detailed discussion of the reasons for choosing some existing results, as followed:

These indexes include 3 primary indexes and 9 secondary indexes. The first fist-level index is the construction level of emergency information sharing mechanism, which has four secondary indexes, including emergency information reporting, emergency information security, and emergency information sharing incentives. The second first-level index is the resource support capability of emergency information sharing, which has two second-level indexes, including the unity of emergency information standards, and financial support. The third first-level index is the collaborative driving force of emergency information sharing, and it has three second-level indexes, including clear management functions, obstacles to emergency information sharing, and the integration ability of emergency management agencies. These indexes can scientifically evaluate the collaborative efficiency of information sharing in coastal cities.
